# Exploration of the spatial patterns and determinants of asthma prevalence and health services use in Ontario using a Bayesian approach

**DOI:** 10.1371/journal.pone.0208205

**Published:** 2018-12-10

**Authors:** Alexandra M. Ouédraogo, Eric J. Crighton, Michael Sawada, Teresa To, Kevin Brand, Eric Lavigne

**Affiliations:** 1 Institute for Clinical Evaluative Sciences, Toronto, Ontario, Canada; 2 Department of Geography, Environment and Geomatics, University of Ottawa, Ottawa, Ontario, Canada; 3 The Hospital for Sick Children, Toronto, Ontario, Canada; 4 Dalla Lana School of Public Health, University of Toronto, Toronto, Ontario, Canada; 5 Telfer School of Management, University of Ottawa, Ottawa, Ontario, Canada; 6 Air Health Science Division, Health Canada, Ottawa, Ontario, Canada; 7 School of Epidemiology and Public Health, University of Ottawa, Ottawa, Ontario, Canada; Western University, CANADA

## Abstract

The purpose of this study was to examine the spatial variability of asthma outcomes in Ontario, Canada and broad environmental factors that contribute to this variability. Age-/sex-standardized asthma prevalence and health services use rates (2003–2013) were obtained from a provincial cohort of asthma patients. Employing an ecological-level study design, descriptive and Bayesian spatial regression analyses were used to examine patterns of asthma outcomes and their relationship to physical environment, socioeconomic environment and healthcare factors. Significant spatial variation in asthma outcomes was found between southern urban/suburban areas and northern/rural areas. Rurality was found to have a substantial effect on all asthma outcomes, except hospitalizations. For example, the most rural areas were associated with lower asthma prevalence and physician visits [RR = 0.708, 95% credible interval (CI): 0.636–0.795 and RR = 0.630, 95% CI: 0.504–0.758, respectively], and with higher ED visits (RR = 1.818, 95% CI: 1.194–2.858), when compared to urban areas. Strong associations were also found between material deprivation and ED visits (RR = 1.559, 95% CI: 1.358–1.737) and hospitalizations (RR = 1.259, 95% CI: 1.143–1.374). Associations between asthma outcomes and environmental variables such as air pollution and temperature were also found. Findings can be expected to inform the development of improved public health strategies, which take into account local environmental, socioeconomic and healthcare characteristics.

## Introduction

Asthma is the most common chronic respiratory disease, affecting an estimated 334 million people globally [1]. Asthma places a considerable burden on affected individuals, the health care system and society as a whole in terms of lost productivity. However, the asthma burden is not spatially homogenous. In fact, in the context of Ontario, Canada, it has been shown that there is a 5-fold variation in asthma prevalence rates across the province [2]. And while risk factors such as genetic predisposition, atopy, infections, diet and second-hand smoke are commonly identified as important individual asthma risk factors [3–5], they do not adequately explain the considerable spatial heterogeneity that has been identified previously [6,7]. Looking beyond individual risk factors, this study uses an ecological level study design and Bayesian spatial analysis approach to examine the potential effect of environmental, socioeconomic and healthcare factors on asthma prevalence and health services use in the context of Ontario, Canada.

There is a vast international body of research on the association between asthma development and exacerbations and individual level risk factors. Studies have shown, for example, strong relationships between asthma outcomes and genetic predisposition [4], exposure to allergens and infections [3,8] and modifiable behavioural factors such as cigarette smoking and diet [5]. Less well understood but increasingly recognized as important, is the role of socioeconomic factors in determining asthma and asthma outcomes [6,9,10]. For example, Gwynn (2004) found higher asthma prevalence among US adults with low socioeconomic status (SES), defined as low education or income, when compared to adults with higher SES. In northern Sweden, Hedlund et al. (2006) found increased risk of developing asthma and respiratory symptoms in manual workers when compared to executives and civil servants, which was mainly attributed to differences in occupational exposures and smoking habits.

To better understand the spatial epidemiology of the illness, many area-level studies have used exploratory analyses based on the simple visualization of standardized mortality/morbidity rates (SMRs) [2,11,12] or non-spatial regression analyses to explore associations with potential covariates [13,14]. The analysis of SMRs is limited by the fact that estimates may be unstable due to overdispersion in disease counts. For example, extreme values may be observed in areas with low or sparse populations. This method also does not account for similarities of disease risk in neighbouring areas (i.e. spatial correlation), which may be due to shared socioeconomic characteristics or environmental exposures [15,16]. In addition, while non-spatial regression analyses can account for potential covariates, they do not account for residual spatial correlation, which can be due to unmeasured confounders or shared characteristics between neighbouring areas. For these reasons, there is an increasing interest in using Bayesian spatial methods to account for overdispersion, unmeasured confounding, and spatial correlation, and thus, to better understand the geographic patterns of health outcomes [17,18]. A number of recent studies have used these methods to describe the spatial and/or temporal trends of asthma and identify at-risk areas [19–23]. For example, Boulieri et al. (2016) found that risk of chronic respiratory diseases was highest in urban northeastern parts of England and suggested this may be due to higher deprivation, air pollution or smoking prevalence in these areas.

This study builds on a previous Ontario study that examined the geographic patterns of asthma prevalence across the province [11]. While findings showed clusters of high rates in many urban and industrial areas and clusters of low rates in suburban and most rural areas, the research did not examine other health care use outcomes or the factors that determine these patterns. This research addresses this gap by examining the spatial patterns of multiple outcomes, including asthma prevalence, physician visits, emergency department (ED) visits and hospitalizations, and by investigating the role of ecological level environmental, socioeconomic and health care factors using a Bayesian spatial approach. Findings can be expected to inform the development of improved public health strategies, which take into account local environmental, socioeconomic and health care characteristics.

## Materials and methods

**Fig 1:**
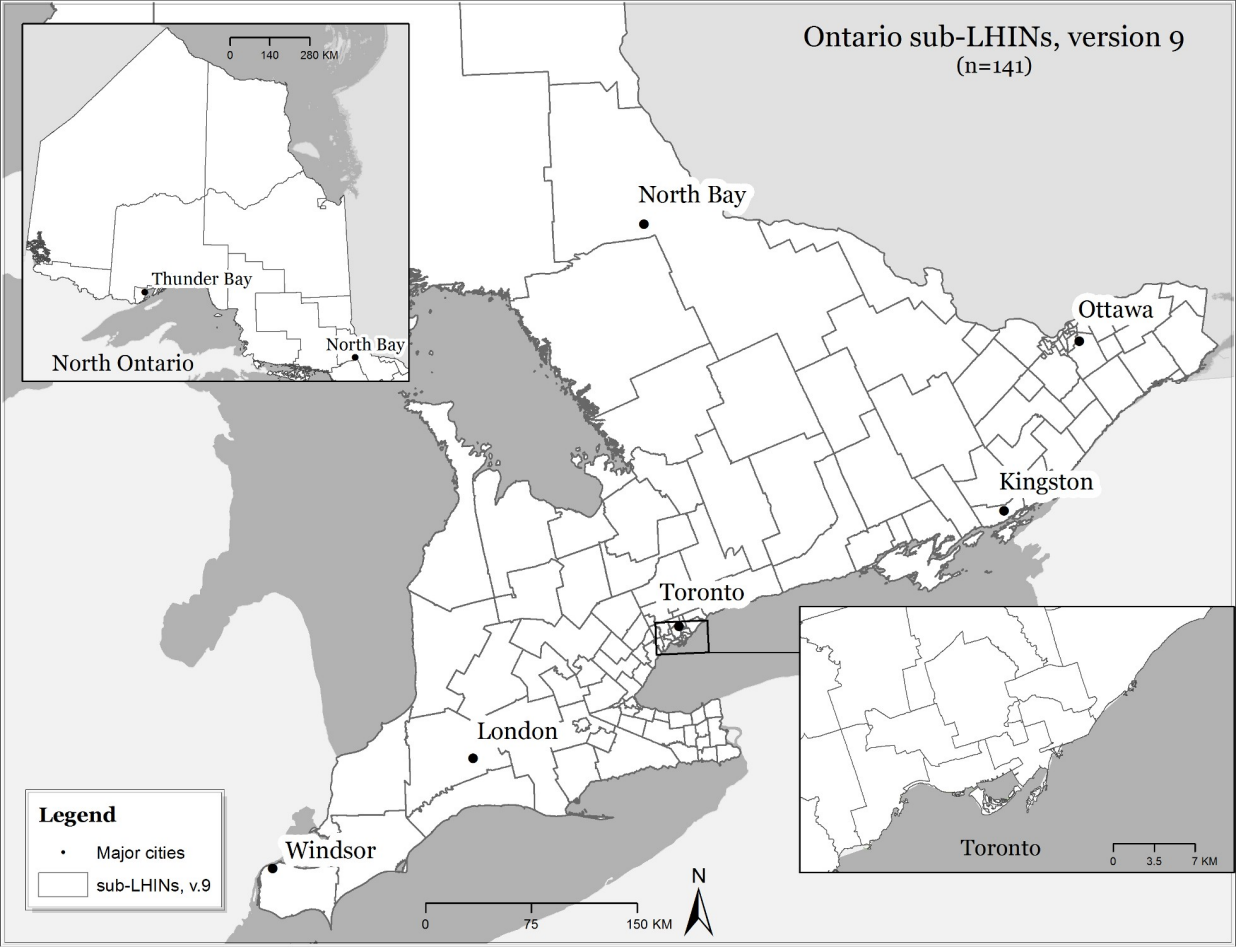
Study area, Ontario sub-Local Health Integration Network (sub-LHINs) (version 9).

We conducted a population-based, ecological-level study to examine the spatial variation of asthma outcomes in the province of Ontario, Canada, and factors that may explain this variation over an 11-year period (2003 to 2013). The geographic unit of analysis used here is the secondary sub-Local Health Integration Network (sub-LHIN). Ontario is divided into 14 LHINs which are subdivided into sub-LHINs (n = 141) for more refined local planning (average population = 12,901) (Fig 1).

Ontario is the most populated province in Canada with approximately 14 million residents [24]. The population’s distribution varies from sparsely populated rural areas in the North to densely populated urban areas located mostly in the South. For residents of Ontario, access to health care services is universal through the Ontario Health Insurance Program (OHIP), although northern and rural residents must frequently travel significantly longer distances to access health services as compared to urban residents [25,26].

### Asthma outcomes

A total of four asthma outcome variables were examined in this research: prevalence, asthma-specific physician visits, ED visits and hospitalizations. This data was obtained through the Ontario Asthma Surveillance Information System Database (OASIS). OASIS is a validated registry of all Ontario residents with asthma (International Classification of Diseases ICD-10 codes: J45, J46), housed at the Institute for Clinical Evaluative Sciences (ICES), an independent, not-for-profit research institute. The registry was generated using: (1) Ontario Health Insurance Plan (OHIP) database, which contains information on billings for physician services; (2) Canadian Institute for Health Information Discharge Abstract Database (CIHI-DAD), which contains information on hospitalizations; (3) National Ambulatory Care Reporting System (NACRS), which contains data on emergency room visits; and (4) Registered Persons Database (RPDB), which contains personal information of individuals registered for OHIP such as age, gender, health card number, and postal code (which determines the residential location per sub-LHIN). These datasets were linked using unique encoded identifiers and analyzed at ICES. Prevalent asthma cases are defined as anyone with at least two asthma-related physician visits within two consecutive years and/or at least one asthma hospitalization since April 1, 1991. This definition, described in detail elsewhere [27,28], yielded 89% sensitivity and 72% specificity in children (ages under 18) and 84% sensitivity and 76% specificity in adults (18 years and over). Due to the evidence that asthma may remit but not resolve, identified individuals with diagnosed asthma remain in the registry until they die or relocate outside of the province.

Average prevalence and health services rates were calculated over the 11-year study period. This method was used to minimize variations due to random fluctuations of small numerators relative their denominators from year to year. Population estimates for all sub-LHINs were not available from any one source, therefore sub-LHIN prevalence rates were calculated using 2009 weighted population data from the RPDB, 2006 population data at a primary sub-LHIN level from the Ministry of Health and Long-term Care, and 2003-to-2013 intercensal and postcensal population estimates at the LHIN level from Statistics Canada. Prevalence rates were calculated by dividing the number of individuals meeting the asthma case definitions by the sub-LHIN population whereas health service use rates were calculated by dividing the number of individuals who used the particular health service (asthma-specific) by the prevalent population. Using the 2009 Ontario population, rates were age and sex standardized (indirect method) and standardized morbidity ratios (SMRs) were then calculated.

### Explanatory variables

The choice of explanatory variables was informed by a population health framework [29] and by the determinants of asthma literature [6,7,9]. Explanatory variables included in the analysis are grouped into three categories: physical environment, socioeconomic environment, and health care access (Table 1). Environmental data was obtained from three different sources and includes pollen concentrations, air pollution concentrations and climate normals. Ontario pollen counts from mid-March to mid-October and covering a five-year period (2006–2011) were obtained from Aerobiology Research Laboratories Inc., Canada. Average total pollen concentration maps for the period 2006–2011, at a 10km resolution, were produced using an inverse distance weighting technique. This data is based on seven pollen-sampling stations across Ontario, primarily located in the southern region, which collect daily concentrations from various types of pollen including grasses, trees and weeds. Due to the limited number of stations, pollen concentration values are considered crude estimates. Postal code-level pollution variables were obtained from Health Canada. These included satellite-derived long-term estimates of air pollution, specifically annual concentration of fine particulate matter with diameter of 2.5 μm or less (PM_2.5_) for the period of 1998–2006, as well as average 2006 nitrogen dioxide land-use regression (NO_2_-LUR) model estimates [30,31]. The 2006 NO_2_-LUR variable was chosen because it was found to produce the most reliable estimates on within-city variability [31]. Finally, climate normals estimates over 30 years for mean annual precipitation, relative humidity (RH) and extreme maximum temperature at 1 km resolution, were obtained from AdaptWest’s spatial database for North America conservation planning in [32]. Environmental exposure variables were created using ArcGIS tools (version 10.2) to average values from variables with higher spatial resolution into sub-LHIN areas based on location.

**Table 1:** Description of explanatory variables.

Variables	Description	Data sources
***Physical environment***		
Pollen	Total pollen concentration for the period 2006–2011 (p/m3)	Aerobiology Inc.
PM_2.5_	Average concentration of fine particulate matter air pollution between 1998–2006 (ug/m3)	Health Canada
NO_2_	Average 2006 Nitrogen dioxide (NO_2_) concentration (ppb)	
Precipitation	Mean annual precipitation over 30 years (mm)	AdaptWest
Maximum temperature	Extreme maximum temperature over 30 years (°C)	
Relative humidity	Mean annual relative humidity over 30 years (%)	
***Socioeconomic environment***		
Material deprivation	% no high school graduation, lone parent families, government transfers, unemployment, low income, homes needing major repairs (standardized scores: e.g. lowest scores = least deprived; highest scores = most deprived)	ON-Marg^a^
Residential instability	% people living alone, youth, persons per dwelling, apartments, married, owner-occupied house, residential mobility in past 5 years (standardized scores)	
Ethnic concentration	% recent immigrants and visible minorities (standardized scores)	
Dependency	% seniors, ratio of population ages 0–14 and 65+ to population ages 15–64, labour force participation (standardized scores)	
***Health care access***		
Physicians	Number of family physicians and general practitioners per 10,000 persons	IPDB^b^
Rurality	Degree of rurality, ranging from 1 (urban/least rural) to 6 (most rural)	AOHC^c^

^a^ON-Marg: Ontario Marginalization index.

^b^IPDB: ICES Physician database.

^c^AOHC: Association of Health Centre.

Socioeconomic variables were drawn from the 2006 census-based and geographically derived, Ontario Marginalization Index (ON-Marg), which measures the level of marginalization in each geographic unit [33,34]. This index was created using principal factor component analysis and contains four dimensions of marginalization, specifically material deprivation, residential instability, ethnic concentration and dependency. Each dimension is expressed in standardized factor scores for each area. A higher factor score represents a higher degree of marginalization. This index has been found to be stable, and associated with various health outcomes [34]. Health care accessibility was approximated using two variables. First, an indicator of physician supply was obtained from ICES Physician database (IPDB), which represent the number of family physicians and general practitioners actively practicing in Ontario per 10,000 persons in 2008, the study period’s mid-point. Second, a measure of the degree of rurality and underserviced areas was obtained from a publicly available report from the Association of Ontario Health Centre (AOHC) [35]. This measure was derived from the percentage of people living in rural areas and the Rurality Index for Ontario, which is a composite measure that takes into account population density and travel time to the nearest health care referral center [36]. It categorizes sub-LHINs into six area types ranging from 1 (urban/least rural) to 6 (most remote and rural).

Initial variable screening involved calculating correlation coefficients and checking for linearity between explanatory and outcome variables. Variance Inflation Factor (VIF) analysis using a step-wise deletion process was then employed to assess the level of multicollinearity and remove variables with the highest VIF value. There were seven remaining explanatory variables included in the models: the physician variable, material deprivation index, maximum temperature, rurality index, NO_2_, RH and total pollen concentration (see Table 2). Continuous explanatory variables went through a deviation from the mean transformation for easier interpretation.

**Table 2:** Summary of selected explanatory variables, at the Ontario sub-LHIN level (n = 141).

	Minimum	25th percentile	Median	75th percentile	Maximum
Deprivation index (scores)	-1.15	-0.47	-0.2	0.05	1.46
Rurality (1–6)	1	1	2	3	6
Relative humidity (%)	56.02	63.84	66.07	67.67	69.56
Maximum temperature (degrees C.)	34.62	36.07	36.69	37.23	37.93
FP/GPs (per 10,000 persons)	0	6.34	8.62	11.4	51.08
NO_2_ level (ppb)	3.64	6.3	8.71	13.02	24.84
Total pollen (p/m3)	118.14	134.99	140.42	141.56	161.27

## Analysis

Exploratory analysis was first conducted to visualize asthma SMRs and determine the degree of spatial autocorrelation in the asthma outcome variables using Moran’s I. Significant positive spatial autocorrelation indicates that nearby asthma SMRs tend to be similar in magnitude within neighbouring sub-LHINs, whereas significant negative spatial autocorrelation would indicate that neighbouring values are systematically different in magnitude. Because the spatial processes driving asthma SMR are unknown, neighbour relationships were defined using the k = 8 nearest neighbours method, which produced similar but more robust results when compared to other methods. Local Indicator of Spatial Autocorrelation (LISA) analysis was then conducted using Local Moran's I to assess the degree of local clustering [37]. LISA analysis allows for both the decomposition of the global indicator into the contribution of each individual observation and also indicates clustering of similarly high or low asthma SMR values as well as spatially outlying asthma SMR values that are high in magnitude surrounded by low values and vice-versa. To test for significant departures from the null hypothesis of no spatial autocorrelation, Monte Carlo simulations with 999 permutations at α<0.05 was used. A Bonferroni correction was applied to account for multiple testing.

Secondly, Poisson log-linear regression models were used to account for the limitations of SMR analyses, e.g. unstable rates due to overdispersion and lack of adjustment for spatialcorrelation, and to examine relationships with the selected explanatory variables. Recognizing that spatial dependence of model residuals represents a violation of Poisson regression assumptions, model residuals were tested for spatial dependence using Moran’s I. To account for spatial autocorrelation in model residuals, spatial random effects were modelled using Conditional Autoregressive (CAR) priors, by borrowing information from neighbouring areas. To do this, we employed Bayesian spatial modelling techniques, specifically generalized linear mixed models (GLMM) for area data. These models were fitted using a hierarchical approach. The first stage of the models specified a likelihood model, representing the distribution of age and sex-adjusted asthma counts in each sub-LHIN. In the second stage, the spatial random effect was specified using the Leroux CAR prior [38], given by

$$\varphi_{i}∼N\left(\frac{\rho \sum_{j=1}^{n}w_{ij}\varphi_{j}}{\rho \sum_{j=1}^{n}w_{ij}+1-\rho },\frac{\tau^{2}}{\rho \sum_{j=1}^{n}w_{ij}+1-\rho }\right),$$

where *φ*_*j*_ is the random effect of neighbouring areas and *w*_*ij*_ is the adjacency matrix parameter, with *w*_*ij*_ = 1 if areas i and j share a common border and *w*_*ij*_ = 0 otherwise. ρ is the spatial autocorrelation parameter, with ρ = 0 representing no spatial autocorrelation and ρ = 1 representing strong spatial autocorrelation, and the conditional variance of the random effect is proportional to τ^2^. A uniform prior distribution on the interval (0,1) is assigned to ρ and an inverse-gamma distribution(shape = 1, scale = 0.01) is assigned to τ^2^.

The Leroux CAR model was chosen as a previous simulation study reported that this model consistently produced good results across a range of spatial correlation scenarios and it was the most theoretically appealing when compared to other random effect models [39]. For Bayesian spatial models, inference was based on Markov Chain Monte-Carlo (MCMC) simulations of a single chain, using a combination of Gibbs and Metropolis sampling methods. Model parameters (i.e. posterior means) were estimated using 500,000 iterations and 100,000 burn-in iterations. Evidence of an association between asthma outcomes and explanatory variables was determined based on whether the 95% credible interval contained zero or not. Model performance was evaluated by plotting parameter samples to visually check for convergence. Spatial modelling was done using the CarBayes package version 4.4 [40] in R (R version 3.1.2). The dataset creation plan and analytic codes used in this study are provided in the supplementary materials (S1 and S2 Text).

### Ethics statement

ICES is a prescribed entity under section 45 of Ontario’s Personal Health Information Protection Act. Section 45 authorizes ICES to collect personal health information, without consent, for the purpose of analysis or compiling statistical information with respect to the management of, evaluation or monitoring of, the allocation of resources to or planning for all or part of the health system. Projects conducted under section 45, by definition, do not require review by a Research Ethics Board. This project was conducted under section 45, and approved by ICES’ Privacy and Compliance Office. All patient records were fully anonymized before the data was accessed by the authors.

## Results

Summary statistics for all asthma outcomes are presented in Table 3. In Ontario, there were 1,818,971 prevalent cases of asthma (141.09 per 1000) identified during the study period, with 641,065 physicians visits (352.43 per 1000), 41,304 ED visits (22.71 per 1000) and 13,635 hospitalizations (7.50 per 1000). Note that the denominator used for asthma prevalence rate is the sub-LHIN population, whereas the denominator used for health services rate is the asthma prevalent population. The highest variability in rates among sub-LHIN areas was seen in the ED visits [Coefficient of variation (CV) = 54%] and hospitalizations (CV = 44%) as compared to physician visits (CV = 23%) and prevalence (CV = 18%).

**Table 3:** Summary of asthma prevalence, physician visits, ED visits and hospitalizations total counts and average rates by geographic level, in Ontario, 2003–2013.

	Province level		sub-LHIN level				
	Counts	Rate (per 1000)	Mean	SD^a^	Minimum	Maximum	CV^b^ (%)
Prevalence	1,818,971	141.09	140.71	24.85	58.38	278.56	17.66
Physician visits	641,065	352.43	311.01	72.85	151.34	517.05	23.42
ED visits	41,304	22.71	28.39	15.38	8.71	76.65	54.16
Hospitalizations	13,635	7.50	7.76	3.39	3.78	38.50	43.66

Note: The denominator used for asthma prevalence rate is the sub-LHIN population, whereas the denominator used for health services rate is the asthma prevalent population.

^a^SD: Standard deviation.

^b^CV: Coefficient of variation.

Maps of SMRs and LISA results for asthma prevalence, physician visits, ED visits and hospitalizations revealed considerable and distinct spatial patterns across Ontario sub-LHINs. Moran’s I tests indicated the presence of moderate but statistically significant global spatial autocorrelation (prevalence: I = 0.40; physician visits: I = 0.64; ED visits: I = 0.52; hospitalizations: I = 0.30; all p-values< 0.05), indicating that asthma outcomes are more similar in neighbouring sub-LHINs than would be expected by chance.

**Fig 2:**
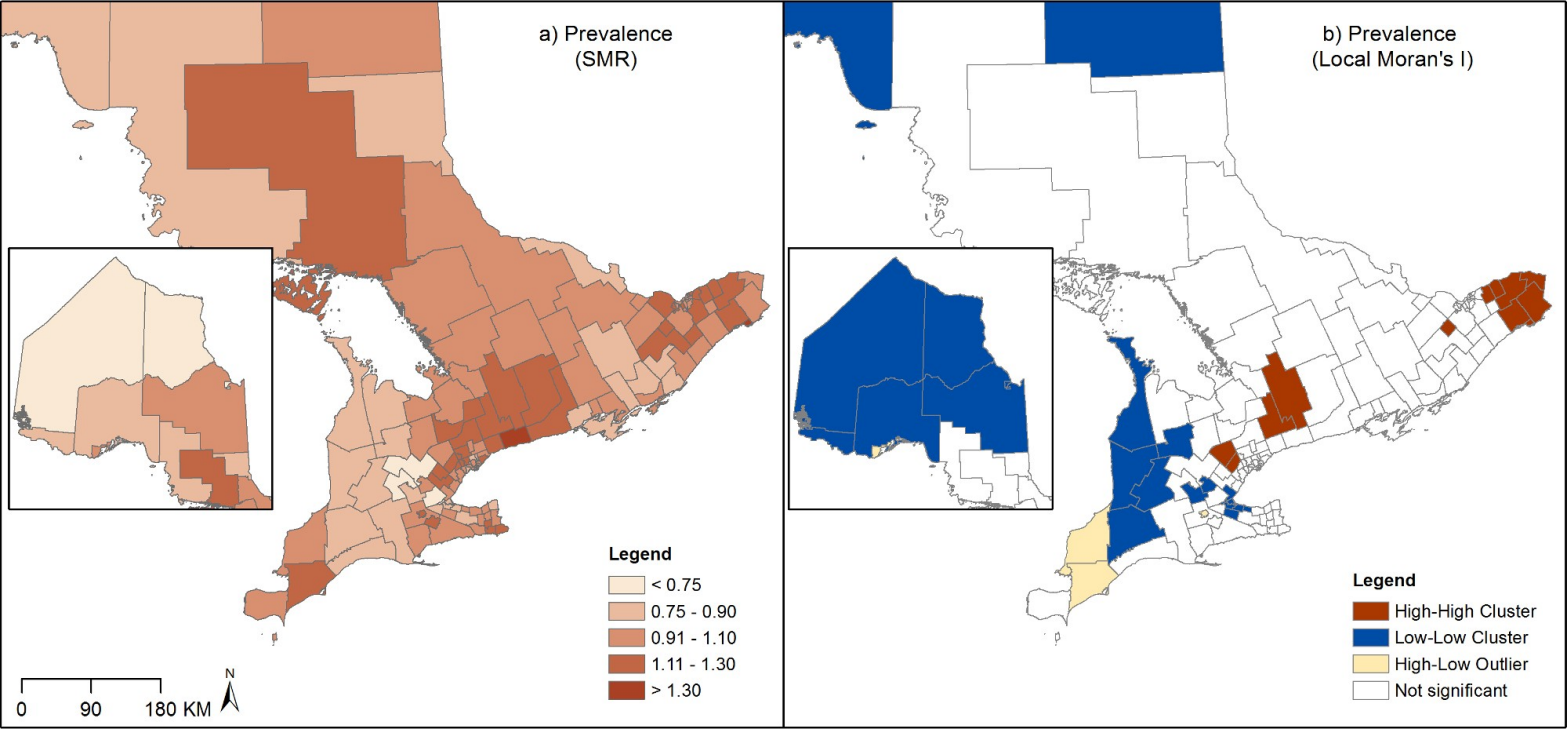
Standardized morbidity ratios (SMRs) and Local Moran's I of asthma prevalence (Ontario, Canada, 2003–2013).

Asthma Prevalence: A 4.9-fold variation was found in asthma prevalence across Ontario, with the highest SMRs in southeastern areas of the province near Ottawa, in southcentral areas near Toronto, and southwestern areas near Windsor, and the lowest SMRs were found in the most northern and rural southern areas. LISA analysis revealed several significant clusters of high SMRs in suburban areas near Toronto and rural areas near Ottawa, and clusters of low SMRs in the North and rural South of the province. One high-low outlier was also identified in southwestern Ontario (Fig 2).

Physician visits: The spatial patterns of asthma physician visits are similar to prevalence, with a 3.4-fold variation between the highest SMRs in the Toronto, Ottawa and Windsor areas, and the lowest SMRs in the North and rural South. LISA analysis identified two clusters of high SMRs in the urban areas of Toronto and Ottawa, and clusters of low SMRs in northern, as well as rural southeastern and southwestern areas. Several high-low outliers were also found in rural southeastern, southwestern and northern areas and two low-high outliers were identified around Toronto area.

**Fig 3:**
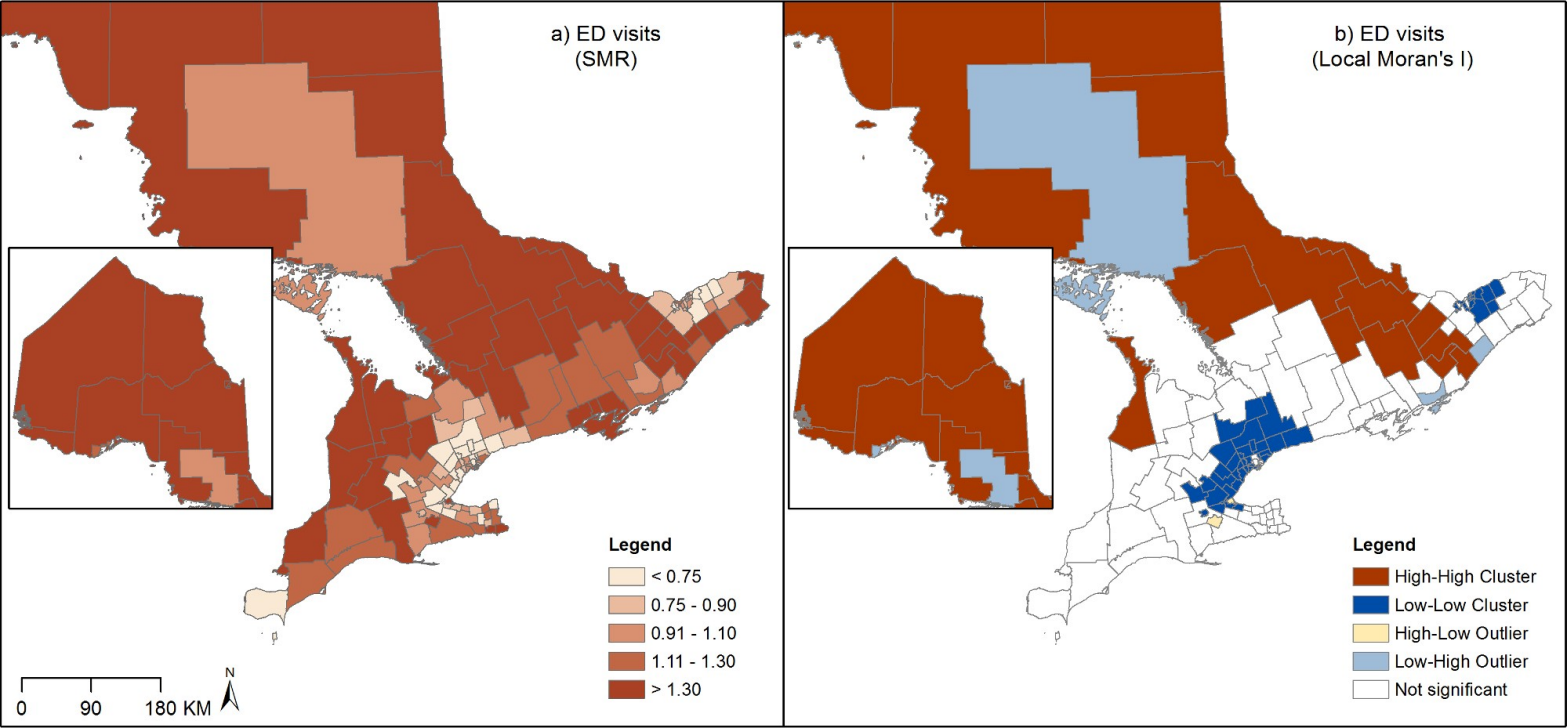
Standardized morbidity ratios (SMRs) and Local Moran's I of ED visits (Ontario, Canada, 2003–2013).

ED visits: The highest SMRs are seen in the north and rural south of the province, whereas lowest SMRs are in and around the urban areas of Toronto and Ottawa, representing a 9-fold variation across the province (Fig 3). Clusters of high SMRs were identified in northern and rural southeastern areas, as well in a rural southwestern area. Two clusters of low SMRs were found in urban/suburban areas in and around Toronto and East of Ottawa. Two high-low outliers were identified in areas west of Toronto whereas several low-high outliers were found in southeastern and northern areas.

Hospitalizations: The largest (9.6-fold) variation across Ontario was found in hospitalization rates. The spatial patterns are similar to ED visits, although the highest SMRs are found mainly in the north of the province, and the lowest SMRs are in suburban areas north of Toronto and west of Ottawa. LISA analysis confirmed these patterns.

**Fig 4:**
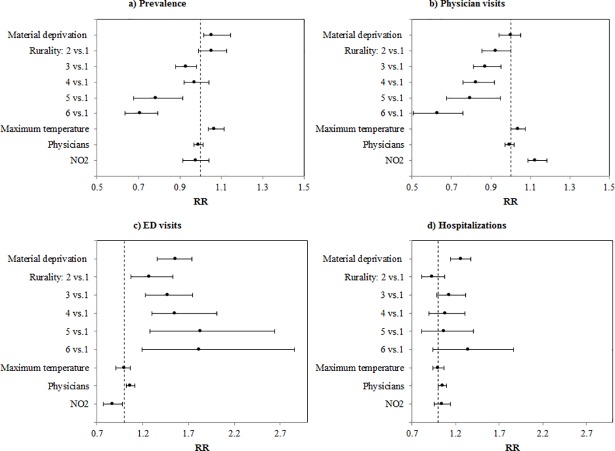
Relative risks (RRs) associated with material deprivation, rurality, NO_2_, maximum temperature and physicians for a) asthma prevalence, b) physician visits, c) ED visits and d) hospitalizations (Ontario, Canada, 2003–2013).

Important findings from the spatial regression models are summarized in Table 4 and Fig 4, which show the relative risk across different asthma outcomes associated with each explanatory variable found. Results revealed that material deprivation, rurality, physicians, maximum temperature and NO_2_ could have substantial effects depending on the outcome examined. Material deprivation increased asthma prevalence by 5.3% (RR = 1.053; 95% CI: 1.015, 1.146), ED visit by 55.9% (RR = 1.559, 95% CI: 1.358,1.737), and hospitalizations by 25.9% (RR = 1.259, 95% CI:1.143,1.374). No significant association was found between material deprivation and physician visits. In contrast, increasing rurality substantially affected the spatial patterns of all asthma outcomes, except hospitalization. For example, while most rural areas were found to be associated with lower asthma prevalence and physician visits, by 29%(RR = 0.708, 95% CI: 0.636, 0.795) and 37% (RR = 0.630, 95% CI: 0.504, 0.758) respectively, they were associated with 81% higher ED visits (RR = 1.818, 95% CI:1.194, 2.858), when compared to urban areas. Findings also suggested a positive association with hospitalizations, but the credible intervals did not provide enough information to draw any conclusion. For the relationship between NO_2_ and asthma outcomes, we found that higher NO_2_ concentration was associated with a decrease in ED visits by 13.2% (RR = 0.868, 95% CI: 0.769,0.974), and an increase in physician visits by 12.5% (RR = 1.125, CI: 1.088, 1.184). Maximum temperature was positively associated with asthma prevalence (RR = 1.067, 95% CI: 1.039,1.113) and physician visits (RR = 1.038, 95% CI: 1.001, 1.075), and the physician variable was also positively associated with ED visits (RR = 1.063, 95% CI: 1.016,1.114) and hospitalizations (RR = 1.048, 95% CI: 1.001,1.096).

**Table 4:** Relative risks associated with explanatory variables and summary of Bayesian variance parameters by asthma outcome (Ontario, Canada, 2003–2013).

	Prevalence	Physician visits	ED visits	Hospitalizations
	RR (95%CI)	RR (95%CI)	RR (95%CI)	RR (95%CI)
Material deprivation	1.053 (1.015,1.146)*	1.001 (0.943,1.052)	1.559 (1.358,1.737)*	1.259 (1.143,1.374)*
Rurality				
1 (urban/least rural)	Reference	-	-	-
2	1.054 (0.989,1.127)	0.924 (0.856,1.003)	1.271 (1.073,1.528)*	0.928 (0.809,1.076)
3	0.929 (0.879,0.98)*	0.871 (0.810,0.952)*	1.471 (1.228,1.744)*	1.129 (0.979,1.314)
4	0.970 (0.919,1.04)	0.824 (0.760,0.917)*	1.551 (1.302,2.006)*	1.082 (0.893,1.306)
5	0.785 (0.676,0.915)*	0.796 (0.676,0.947)*	1.833 (1.275,2.641)*	1.066 (0.812,1.402)
6 (most rural/remote)	0.708 (0.636,0.795)*	0.63 (0.504,0.758)*	1.818 (1.194,2.858)*	1.344 (0.94,1.865)
Relative humidity	0.936 (0.874,0.983)*	0.965 (0.910,1.024)	1.043 (0.939,1.198)	0.892 (0.816,0.976)*
Maximum temperature	1.067 (1.039,1.113)*	1.038 (1.001,1.075)*	0.997 (0.906,1.066)	0.998 (0.939,1.062)
Physicians	0.990 (0.969,1.012)	0.994 (0.970,1.019)	1.063 (1.016,1.114)*	1.048 (1.001,1.096)*
NO_2_	0.978 (0.915,1.040)	1.125 (1.088,1.184)*	0.868 (0.769,0.974)*	1.042 (0.951,1.137)
Pollen	1.026 (0.997,1.061)	1.035 (0.989,1.082)	0.927 (0.856,1.027)	1.060 (0.978,1.15)
** **	**Posterior mean (95%CI)**	**Posterior mean (95%CI)**	**Posterior mean (95%CI)**	**Posterior mean (95%CI)**
Spatial variance, τ^2^	0.047 (0.033,0.065)	0.037 (0.024,0.055)	0.159 (0.103,0.245)	0.067 (0.037,0.107)
Spatial correlation, ρ	0.657 (0.368,0.930)	0.531 (0.248,0.846)	0.406 (0.160,0.73)	0.518 (0.147,0.89)

*95% credible interval did not contain zero.

CI: credible intervals.

While the association estimates of the spatial models were similar to the models with no random effects (S1 Table), the spatial models accounted for spatial dependence in all the models. Moderate to large spatial autocorrelation was accounted by the models, with posterior estimates for ρ ranging between 0.406 in ED model and 0.657 in prevalence model. The spatial random effect variance also varied across the outcomes (i.e. posterior estimates for τ^2^ ranging between 0.037 in physician visits model and 0.159in ED visits model) (Table 4). Global Moran’s I tests and residual maps also confirmed that no significant spatial autocorrelation remained in the models, after accounting for spatial autocorrelation.

## Discussion

The goal of this study was to examine the spatial patterns of prevalence and health services use for physician diagnosed asthma, and to better understand the role of ecological determinants in explaining these patterns. Significant spatial patterns in asthma outcomes were found across Ontario. For asthma prevalence, clusters of high SMRs were found in suburban areas near Toronto and in rural areas near Ottawa, whereas clusters of low SMRs were located in the north and rural south of the province. Conversely, the variation of asthma ED visits and hospitalizations showed the opposite pattern; large clusters of high rates were found in most northern and rural areas in the south of the province, whereas clusters of low SMRs were found in areas near Toronto. Similar urban/rural patterns have been reported in previous Ontario studies examining the spatial patterns of asthma [2,11] suggesting that differences in health care access and environmental exposure may be at play in determining these patterns.

Our models revealed that while most rural areas were associated with lower asthma prevalence and physician visits, they were associated with higher ED visits, when compared with urban areas. Lower asthma prevalence in rural areas compared to urban areas has been previously reported in the literature [6,41]. In these studies, the patterns are explained by an increased likelihood of diagnosis in urban areas due to better access and more regular contact with primary care physicians, combined with urban risks factor including higher levels of outdoor air pollution. A further potential explanation can be found in the hygiene hypothesis which posits that increased early life exposure to germs (from farm environment, large family households, etc.) is associated with reduced likelihood of developing asthma [42,43]. In contrast, higher ED visit and hospitalization rates in rural, northern or remote areas may be explained by reduced access to primary physicians resulting in reliance on emergency services for basic health care or poorer asthma management, resulting in more severe reactions [25].

Material deprivation was moderately associated with asthma prevalence, but strongly associated with ED visits and hospitalizations. The positive association between SES and ED visits and hospitalizations has been previously reported in the literature [44,45], where it is suggested that this may be due to inadequate access and use of asthma medications. Moreover, the moderate association found between material deprivation and asthma prevalence may reflect poorer health status in lower SES groups, which may be related to higher stress, poorer housing conditions or presence of comorbidities [9]. It may also be explained by an increased likelihood of being diagnosed due to frequent health services use.

Maximum temperature was also found to have a weak but positive association with asthma prevalence and physician visits. This is consistent with the growing evidence of an indirect effect of extreme temperatures on asthma outcome, via complex environmental processes such as the modification of production and dispersion of allergen or increased pollution levels, which may trigger asthma symptoms [46,47]. More sophisticated temporal models are needed to account for the seasonal and temporal trends of these variables to further explain this association.

While both PM_2.5_ and NO_2_ pollution variables were examined in a preliminary multicollinearity analysis (results not shown), only NO_2_ was found to be significantly associated with asthma outcomes, and therefore included in the models. NO_2_ was positively associated with asthma physician visits but negatively associated with ED visits. In the case of physician visits, findings are consistent with previous studies which have reported a positive effect of exposure to ambient air pollutants, including NO_2_, particulate matter (PM), ozone (O_3_) and carbon oxides (CO), on asthma exacerbations and increased use of health services [48,49]. The negative relationship to ED visits is inconsistent with the literature; however, it could be explained by the fact that NO_2_ may be lower in areas with high asthma ED visits, which were found to be mainly northern and rural areas. Another explanation could be that the NO_2_-LUR variable used in this study does not adequately capture the spatial variability of air pollution, as this variable was found to contain less uncertainty in urban areas where most monitors are located as compared to rural areas [31]. Better air pollutant measures and/or the effect of other air pollutants, such as O_3_ or CO, should be further examined.

It is also worth noting that a number of variables examined here did not have a substantial impact on asthma outcomes despite evidence of their effects in the literature. While we previously suggested that better access to primary care may increase the likelihood of being diagnosed in urban areas, the physician variable was not associated with asthma prevalence or physician visits. It was however, positively associated with ED visits and hospitalizations. This is inconsistent with the literature since previous studies have reported that better access to primary care physicians and specialists is associated with better asthma management and reduced likelihood of patients experiencing severe symptoms that require emergency care [50,51]. Possible explanations for our findings could be that the physician variable is not a good indicator of primary care accessibility or that the scale and spatial configuration of area units may confound the results. In fact, area-based measures, such as the physician rate, have been found to be limited in their ability to adequately characterize spatial accessibility as they do not account for travel across unit boundaries [52]. The pollen variable was also not significantly associated with any asthma outcome, although the effect of aeroallergen exposure on asthma symptom exacerbations has been shown in the literature at the individual level [53,54]. In this study, non-significant results are mainly explained by the small number of sampling stations across Ontario, which were mainly located in the southern part of the province. This pollen data was based on only seven sampling stations, which is insufficient to properly interpolate over a study area as large as Ontario and thus, interpolated values may be biased in places that are far away from the stations. Better pollen measures would be needed to further investigate this relationship at the ecological level.

There are a few limitations to this research that should be discussed. Firstly, the ecological study design used here does not allow us to make inferences about the determinants of asthma at the individual level. It does however, provide contextual explanations for understanding outcomes across geographies, which has useful implications for the development of public health strategies and interventions. Secondly, the modifiable areal unit problem (MAUP) [55] represents a potential limitation in that the patterns identified here may be dependent on the spatial aggregations used in the creation of sub-LHINs. Testing for this is challenging due to population size constraints at larger scales. Thirdly, while the prevalence measure has been validated and shown to have good sensitivity and specificity for diagnosed cases [27,28], it may be underestimated due to a lack of medication data and less severe cases may go undiagnosed, particularly in rural and northern areas due to poorer access to primary care and diagnostic services. In addition, there may also be a number of unknown or unmeasured ecological risk factors that were not accounted for in this study, including other measures of SES, environmental exposure and access. Finally, the results of any spatial analysis are dependent on the definition of spatial dependency used. Given that there is little understanding of the spatial processes driving asthma SMRs in Ontario, in this study, we choose nearest neighbours as the means of summarizing spatial interaction between sub-LHINs for spatial analytical purposes. In future research, with more information, more appropriate measures of spatial interaction between sub-LHINs (e.g., road network connectivity, travel time etc.) [56] could be used and may provide different results.

This research highlights the utility of large and sophisticated health administrative databases for asthma identification and surveillance in a large population. Also, the use of a Bayesian spatial approach provided a sophisticated and flexible framework, which facilitated the adjustment for various confounders and spatial dependence in the data, and a better understanding of the relationships between asthma outcomes and ecological risk factors. Results demonstrated the presence of significant spatial patterns in asthma outcomes across Ontario, which were mainly explained by material deprivation and rurality. These findings can inform the development of better, locally relevant public health strategies directed towards improving access to health care in rural/northern areas and health outcomes in areas of low SES. Future research should consider more refined measures of air pollution, aeroallergens, physician accessibilityand spatial interaction and include the use of spatio-temporal models to account for time-dependent factors.

## Supporting information

S1 Table Relative risks associated with explanatory variables for each asthma outcome, using models with no spatial random effect (rho = 0) (Ontario, Canada, 2003–2013). (DOCX)

S1 Text Dataset creation plan. (DOCX)

S2 Text Analytic codes. (DOCX)
