# Supplementary material for: Exploration of the spatial patterns and determinants of asthma prevalence and health services use in Ontario using a Bayesian approach
Source: PLoS One. 2018 Dec 10;13(12):e0208205. doi: 10.1371/journal.pone.0208205 (PMC6287847; doi:10.1371/journal.pone.0208205)
Supplement: S1 Table — (DOCX) [file pone.0208205.s001.docx]

**S1 Table. Relative risks associated with explanatory variables for each asthma outcome, using models with no spatial random effect (rho=0) (Ontario, Canada, 2003-2013).**

|  | **Prevalence** | **Physician visits** | **ED visits** | **Hospitalizations** |
| --- | --- | --- | --- | --- |
|  | **RR (95%CI)** | **RR (95%CI)** | **RR (95%CI)** | **RR (95%CI)** |
| Material deprivation | 1.076 (1.025,1.147)* | 0.988 (0.927,1.065) | 1.562 (1.385,1.809)* | 1.303 (1.192,1.428)* |
| Rurality |  |  |  |  |
| 1 (least rural) | Reference | - | - | - |
| 2 | 1.066 (0.996,1.2) | 0.93 (0.832,1.007) | 1.265 (1.055,1.492)* | 0.963 (0.822,1.12) |
| 3 | 0.918 (0.858,0.969)* | 0.894 (0.819,1.007) | 1.456 (1.195,1.74)* | 1.157 (1,1.347)* |
| 4 | 0.978 (0.895,1.065) | 0.85 (0.754,0.94)* | 1.616 (1.293,1.958)* | 1.09 (0.891,1.323) |
| 5 | 0.765 (0.655,0.859)* | 0.773 (0.643,0.936)* | 2.018 (1.425,2.918)* | 1.254 (0.925,1.654) |
| 6 (most rural ) | 0.631 (0.535,0.751)* | 0.65 (0.528,0.781)* | 1.978 (1.394,2.765)* | 1.642 (1.177,2.3)* |
| Relative humidity | 0.925 (0.897,0.968)* | 0.955 (0.905,1.001) | 1.076 (0.963,1.166) | 0.931 (0.867,1.004) |
| Maximum temperature | 1.044 (1.021,1.074)* | 1.034 (1.01,1.058)* | 0.994 (0.94,1.043) | 0.989 (0.94,1.036) |
| Physicians | 0.959 (0.934,0.99)* | 0.991 (0.967,1.016) | 1.075 (0.995,1.147) | 1.048 (1.004,1.096)* |
| NO_2_ | 0.995 (0.972,1.025) | 1.14 (1.084,1.198)* | 0.865 (0.784,0.933)* | 1.051 (0.982,1.123) |
| Pollen | 1.035 (1,1.058) | 1.03 (0.991,1.062) | 0.929 (0.856,0.992)* | 1.05 (0.99,1.107) |
|  | **Posterior mean (95%CI)** | **Posterior mean (95%CI)** | **Posterior mean (95%CI)** | **Posterior mean (95%CI)** |
| Spatial variance, τ^2^ | 0.021 (0.017,0.027) | 0.017 (0.013,0.022) | 0.08 (0.061,0.107) | 0.029 (0.020,0.042) |
| Spatial correlation, ρ | 0 | 0 | 0 | 0 |
