# Supplementary material for: Exploration of the spatial patterns and determinants of asthma prevalence and health services use in Ontario using a Bayesian approach
Source: PLoS One. 2018 Dec 10;13(12):e0208205. doi: 10.1371/journal.pone.0208205 (PMC6287847; doi:10.1371/journal.pone.0208205)
Supplement: S1 Text — (DOCX) [file pone.0208205.s002.docx]

**S1 Text. Dataset Creation Plan.**

**Exploration of the spatial patterns and determinants of asthma prevalence and health services use in Ontario using a Bayesian approach (2018)**

| **Project Objectives:** | 1. Examine spatial patterns of asthma prevalence and health services use in Ontario. 2. Examine the spatial relationship between asthma and physical environment, socioeconomic environment and healthcare factors. | | |  |
| --- | --- | --- | --- | --- |
| ICES Data | | |  | |
| ***General use Datasets - other*** | | ***Rationale*** | ***Years (where applicable)*** | |
| ASTHMA (derived cohort) | | Cohort creation | 2003-2014 | |
| CIHI-DAD | | Cohort creation | 2003-2014 | |
| NACRS | | Cohort creation | 2003-2014 | |
| OHIP | | Cohort creation | 2003-2014 | |
| ***General Use Datasets – Coding/Geography*** | |  |  | |
| LHIN/sub-LHIN | | Cohort stratification | Version 9 | |
| ***General Use Datasets – Population*** | |  |  | |
| RPDB | | Cohort creation/Denominator | 2003-2014 | |
| Statistics Canada/MOHTLC population estimates | | Denominator | 2003-2014 | |
| ***General Use Datasets – Care Providers*** | |  |  | |
| IPDB | | Predictor | 2008 | |
| Other sources | | |  | |
| ON-MARG | | Predictor | 2006 | |
| Health Canada (PM2.5) | | Predictor | 1998-2006 | |
| Health Canada (NO2) | | Predictor | 2006 | |
| Aerobiology Research Laboratories Inc., Canada (pollen) | | Predictor | 2006-2011 | |
| AdaptWest spatial database (climate) | | Predictor | Climate normals (most recent) | |
| Association of Health Centre/Rurality Index of Ontario | | Predictor | 2008 | |

| Project Cohort | | | |  |
| --- | --- | --- | --- | --- |
| **Study Design** | 〼 Cohort study ☐ Matched cohort study ☐ Case-control study  ☐ Cross-sectional study 〼 Other (specify): Ecological study | | |  |
| **Index Event / Inclusion Criteria** | - **Prevalent cases of asthma**   - Using validated algorithm**:** anyone with at least two asthma-related physician visits within two consecutive years and/or at least one asthma hospitalization since April 1, 1991 (see references below) - **Health services use (hospitalization, ED visits, and physician visits) for asthma** using the ICES asthma cohort   - A hospital discharge with any diagnostic code for asthma. Do not count transfers.   - An ED discharge with any diagnostic code for Main problem: asthma. If a patient is transferred from ED to hospitalization with the same disease, only the CIHI hospitalization will be counted.   - A physician office visits with a diagnostic code for asthma. Count one visit per physician per service day per patient   ICD10 codes for asthma: J45, J46  **References:**  *Gershon, A.S.; Wang, C.; Guan, J.; Vasilevska-Ristovska, J.; Cicutto, L.; To, T. Identifying patients with physician-diagnosed asthma in health administrative databases, Can. Respir. J., 2009; Volume 16, pp. 183–188.*  *To, T.; Dell, S.; Dick, P.T.; Cicutto, L.; Harris, J.K.; MacLusky, I.B.; Tassoudji, M. Case verification of children with asthma in Ontario, Pediatr. Allergy Immunol., 2006; Volume 17, pp. 69–76* | | |  |
| **Exclusions (in order)** | *Step* | | Description |  |
|  | 1 | | Missing age, sex |  |
|  | 2 | | Non-Ontario residents |  |
| Project Time Frame Definitions Look-back Window  Observation Window  (in which to look for outcomes)  **Index Event Date**  Accrual Window  Max Follow-up Date | | | | |
|  | | | |  |
| **Accrual Start/End Dates** | | 04/01/2003 – 03/31/2014 | |  |
| **Max Follow-up Date** | | 31/03/2014 | |  |
| **When does observation window terminate?** | | 31/03/2014 | |  |
| **Lookback Window(s)** | | n/a | |  |

| Variable Definitions (add additional rows as needed) | | | |
| --- | --- | --- | --- |
| **Predictors** | - Total pollen concentration - Average concentration of fine particulate matter (PM2.5) - Average 2006 Nitrogen dioxide (NO_2_) concentration - Mean annual precipitation - Extreme maximum temperature - Mean annual relative humidity - Marginalization indices: Material deprivation, Residential instability, Ethnic concentration, Dependency - Number of family physicians and general practitioners per 10,000 persons - Degree of rurality   **Note:**  - Environmental variables are summarized by sub-LHIN using ArcGIS (v.10.2) by averaging values with higher resolutions.  - All predictors are summarized by sub-LHIN. | |  |
| **Primary Outcome Definition** | - Asthma prevalence rates by sub-LHIN | |  |
| **Secondary Outcome Definition(s)** | - Asthma physician visits rates by sub-LHIN - Asthma ED visits rates by sub-LHIN - Asthma hospitalizations rates by sub-LHIN | |  |
| **Baseline Characteristics** | n/a | |  |
| **Other Variables** | - Ontario population by sub-LHIN   - Use 2009 weighted population data (RPDB), 2006 population data at primary sub-LHIN level (from MOHLTC), and 2003-2013 intercensal and post-censal population estimates at the LHIN level (Statistics Canada) - **Denominator for health services use:** asthma prevalent population | |  |
|  | | |  |
| Analysis Plan and Dummy Tables | | |  |
| Step 1:Cohort build  - 1. Apply inclusion and exclusions criteria   2. Build cohort:      1. Get all prevalent asthma cases by sub-LHIN      2. Get all health services use (physician visits, ED visits, Hospitalizations) by sub-LHIN      3. Calculate counts by year, and total counts across years | | |  |
| **Step 2: Outcomes**   - 1. Define outcomes: calculate crude rates      1. Asthma prevalence rates by age, sex, year - Numerator: number of individuals meeting the asthma case definitions - Denominator: Ontario population by sub-LHIN   - 1. Physician visits, ED visits, Hospitalizations rates: - Numerator: number of individuals who used the particular health service (asthma-specific) - Denominator: asthma prevalent population by sub-LHIN   1. Calculate age and sex standardized (indirect method) and standardized morbidity ratios (SMRs), using the 2009 Ontario population | |  |  |
| Step 3: Analysis  - 1. Exploratory analysis:      1. Moran's I test for spatial autocorrelation, using nearest neighbours method.      2. Local Indicator of Spatial Autocorrelation (LISA) analysis to test the degree of local clustering.      3. Test significant departure from null hypothesis using Monte Carlo simulations with 999 permutations and apply Bonferroni correction to account for multiple testing.      4. Produce maps of SMRs and LISA outputs, using ArcGIS (v.10.2)      5. Conduct VIF analysis to test for multicollinearity and remove variables with the highest VIF value.   2. Poisson log-linear regression models, using GLMM and Bayesian approach      1. **Primary analysis:** run models with spatial autocorrelation   - Random effects are modelled using Leroux CAR models  - The spatial autocorrelation parameter (rho) is specified in the model  - Inference is based on Markov Chain Monte-Carlo (MCMC) simulations, using 500,000 iterations  - Analysis is conducted in R, using CARBAYES package (v.4.4) (see reference below)   - - 1. **Sensitivity analysis:** re-run models, without spatial autocorrelation (rho=0)   **Reference:**  *Lee, D.CARBayes: an R package for Bayesian spatial modeling with conditional autoregressive priors, J. Stat. Softw., 2013; Volume 55, pp. 1–24.* | |  |  |
|  | | |  |
