# Supplementary material for: Exploration of the spatial patterns and determinants of asthma prevalence and health services use in Ontario using a Bayesian approach
Source: PLoS One. 2018 Dec 10;13(12):e0208205. doi: 10.1371/journal.pone.0208205 (PMC6287847; doi:10.1371/journal.pone.0208205)
Supplement: S2 Text — (DOCX) [file pone.0208205.s003.docx]

**S2 Text. Analytic codes.**

**Exploration of the spatial patterns and determinants of asthma prevalence and health services use in Ontario using a Bayesian approach**

November 18, 2018

Description

This appendix describes the methods used in the study "Exploration of the spatial patterns and determinants of asthma prevalence and health services use in Ontario using a Bayesian approach" and includes all R codes used in the spatial analysis. The data referenced is held securely in coded form at ICES and access may be granted to those who meet pre-specified criteria for confidential access, available at www.ices.on.ca/DAS. The codes provided may be reused using other outcomes. The paths strings should be modified accordingly.

Part 1: Exploratory analyses

- **Load libraries**

## For general spatial analyses

library(shapefiles)

library(sp)

library(spdep)

library(rgdal)

library(maptools)

library(ggplot2)

## For LISA analysis

library(boot)

library(deldir)

library(foreign)

library(grid)

library(Matrix)

library(nlme)

library(splines)

library(car)

- **Load data**

## Set working directory

setwd("~/Documents/Data")

## Load data

prev.data<-read.csv("prev.csv")

Phys.data<-read.csv("doc.csv")

ed.data<-read.csv("ed.csv")

hosp.data<-read.csv("hosp.csv")

sublhins<-read.csv("empty.csv") #empty dataset with 1 column for sublhin ids

## Load shapefile

poly=readOGR("sublhin_prj.shp")

- **Creating spatial weights/matrix**

To create spatial weights, we use the nearest neighbour definition, but other definitions

can be tested (e.g. queen contiguity method)

sublhinAdjMat= poly2nb(poly, queen=TRUE, snap=0.05) # create adjacency matrix

coords = coordinates(poly) # get coordinates

datknn = knearneigh(coords, k = 8) # create neighbours object

nb = knn2nb(datknn) # create neighbour list

summary(nb)

W.list <-nb2listw(nb, style = "W", zero.policy = FALSE) # create spatial weights for neighbours lists

W.mat<-nb2mat(sublhinAdjMat, style="B") #create spatial weight matrix

- **Run Moran's I tests for residuals, using non-spatial models**

The Moran's I analysis is used to test the presence of spatial autocorrelation in the model residuals. If tests indicate presence of spatial autocorrelation in residuals, spatial models (accounting for spatial random effect) would be needed.

formula.P <- paste("Obs ~ offset(log(Exp)) + depriv_scor + factor(rural) + RH + temp + FPrate + NO2 + tpollen")

## Run GLM with quasipoisson distribution

Prev_nonsp<-glm(formula = formula.P, family = "quasipoisson", data= prev.data)

Phys_nonsp<-glm(formula = formula.P, family = "quasipoisson", data= Phys.data)

ED_nonsp<-glm(formula = formula.P, family = "quasipoisson", data= ed.data)

Hosp_nonsp<-glm(formula = formula.P, family = "quasipoisson", data= hosp.data)

## Summarize

summary(Prev_nonsp); summary(Doc_nonsp); summary(ED_nonsp);summary(Hosp_nonsp)

## Test residuals Spatial Autocorrelation (Moran's I)

resid.Prev_nonsp <-residuals(Prev_nonsp)

resid.Phys_nonsp <-residuals(Phys_nonsp)

resid.ED_nonsp <-residuals(ED_nonsp)

resid.Hosp_nonsp <-residuals(Hosp_nonsp)

moran.mc(x=resid.Prev_nonsp, listw=W.list, nsim=1000)

moran.mc(x=resid.Phys_nonsp, listw=W.list, nsim=1000)

moran.mc(x=resid.ED_nonsp, listw=W.list, nsim=1000)

moran.mc(x=resid.Hosp_nonsp, listw=W.list, nsim=1000)

- **Run local Moran's I using conditional randomization for each location**

The LISA analysis using local Moran's I is conducted to assess the degree of local clustering. This analysis allows for both the decomposition of the global indicator into the contribution of each individual observation and also indicates clustering of similarly high or low asthma SMR values as well as spatially outlying asthma SMR values that are high in magnitude surrounded by low values and vice-versa. The following function was created to undertake Monte Carlo simulation for Local Moran's I at each location. Final maps from this analysis were created using ArcGIS.

## Create function (li.mca) for local Moran's I test

li.mca = function(x,

lw,

nsim = 99,

alternative = "greater"){

n = length(x)

p = vector(length = n)

ml = vector(length = n)

for (i in 1:length(x)){

res = numeric(length = nsim +1)

for (j in 1:nsim) {

xp = x

xp[-i] = sample(x[-i])

xx = mean(xp)

z = xp - xx

lz = lag.listw(lw,z)

s2 = sum(z^2)/(n-1)

res[j] = ((z/s2)* lz)[i]

}

xx = mean(x)

z = x - xx

lz = lag.listw(lw,z)

s2 = sum(z^2)/(n-1)

res[nsim + 1] = ((z/s2) * lz)[i]

#MS: added creates local moran's I values

ml[i]= res[nsim + 1]

rankres = rank(res)

xrank = rankres[length(res)]

diff = nsim - xrank

diff = ifelse(diff > 0, diff, 0)

if (alternative == "less")

pval = punif((diff + 1)/(nsim + 1), lower.tail = FALSE)

else if (alternative == "greater")

pval = punif((diff + 1) / (nsim + 1))

if (!is.finite(pval) || pval<0 || pval>1)

warning("Out-of-range p-value")

statistic = res[nsim + 1]

names(statistic) = "statistic"

parameter = xrank

names(parameter)= "observed rank"

p[i] = pval

}

#MS: added cluster test

#Get cluster quads

cDV = as.vector(scale(x))

c_ml = as.vector(scale(lag.listw(lw,x)))

cDVmean = mean(cDV)

miMean = mean(c_ml)

quadrant = vector(mode = "character", length = length(ml))

quadrant[cDV > cDVmean & c_ml > miMean] = "HH"

quadrant[cDV < cDVmean & c_ml > miMean] = "LH"

quadrant[cDV > cDVmean & c_ml < miMean] = "HL"

quadrant[cDV < cDVmean & c_ml < miMean] = "LL"

# set a statistical significance level for the local Moran's

signif = 0.05

# places non-significant Moran's in the category "5"

quadrant[p > signif] = "NOTSIG"

# Return the moran's local value, p value and cluster tag

return(cbind(ml, p, quadrant))

}

## Add SMRs to shapefile

poly@data$Prev_smr <- prev.data$smr

poly@data$Phys_smr <- phys.data$smr

poly@data$ED_smr <- ed.data$smr

poly@data$Hosp_smr <- hosp.data$smr

dat_prev = poly$Prev_smr

dat_phys = poly$Phys_smr

dat_ed = poly$ED_smr

dat_hosp = poly$Hosp_smr

#### 1) Prevalence (NOTE: save outputs before re-runing for other outcomes)

outputShapeFileName = "~/Documents/Data/output_prev"

## Create conditional simulation for local moran's I with 999 simulation

anres =

li.mca(dat_prev,

lw,

nsim = 999,

alternative = "greater") # Check 'less' alternative as well

## Save outputs

poly$LocalI = anres[,1]

poly$pval = anres[,2]

poly$quad = anres[,3]

writePolyShape(poly, outputShapeFileName)

# Export 8 neighbour LISA results

output_prev=readShapePoly("~/Documents/Data/output_prev", IDvar="SAUID_S")

PrevCheck8nb<-read.csv("empty.csv") # pre-existing empty dataset with sublhin id column

PrevCheck8nb$prev_LocalI <- output_prev$LocalI

PrevCheck8nb$prev_pval <- output_prev$pval

PrevCheck8nb$prev_quad <- output_prev$quad

write.csv(PrevCheck8nb, file="~/Documents/Data/Checking8nb_Prev.csv")

#### 2) Physician visits

outputShapeFileName = "~/Documents/Data/output_phys"

## Create conditional simulation for local moran's I with 999 simulation

anres =

li.mca(dat_phys,

lw,

nsim = 999,

alternative = "greater") # Check 'less' alternative as well

## Save outputs

poly$LocalI = anres[,1]

poly$pval = anres[,2]

poly$quad = anres[,3]

writePolyShape(poly, outputShapeFileName)

# Export 8 neighbour LISA results

output_phys=readShapePoly("~/Documents/Data/output_phys", IDvar="SAUID_S")

physCheck8nb<-read.csv("empty.csv")

physCheck8nb$phys_LocalI <- output_phys$LocalI

physCheck8nb$phys_pval <- output_phys$pval

physCheck8nb$phys_quad <- output_phys$quad

write.csv(physCheck8nb, file="~/Documents/Data/Checking8nb_phys.csv")

#### 3) ED visits

outputShapeFileName = "~/Documents/Data/output_ED"

## Create conditional simulation for local moran's I with 999 simulation

anres =

li.mca(dat_ED,

lw,

nsim = 999,

alternative = "greater") # Check 'less' alternative as well

## Save outputs

poly$LocalI = anres[,1]

poly$pval = anres[,2]

poly$quad = anres[,3]

writePolyShape(poly, outputShapeFileName)

# Export 8 neighbour LISA results

output_ED=readShapePoly("~/Documents/Data/output_ED", IDvar="SAUID_S")

EDCheck8nb<-read.csv("empty.csv")

EDCheck8nb$ED_LocalI <- output_ED$LocalI

EDCheck8nb$ED_pval <- output_ED$pval

EDCheck8nb$ED_quad <- output_ED$quad

write.csv(EDCheck8nb, file="~/Documents/Data/Checking8nb_ED.csv")

#### 4) Hospitalizations

outputShapeFileName = "~/Documents/Data/output_Hosp"

## Create conditional simulation for local moran's I with 999 simulation

anres =

li.mca(dat_Hosp,

lw,

nsim = 999,

alternative = "greater") # May need to check 'less' alternative as well

## Save outputs

poly$LocalI = anres[,1]

poly$pval = anres[,2]

poly$quad = anres[,3]

writePolyShape(poly, outputShapeFileName)

# Export 8 neighbour LISA results

output_Hosp=readShapePoly("~/Documents/Data/output_Hosp", IDvar="SAUID_S")

HospCheck8nb<-read.csv("empty.csv")

HospCheck8nb$Hosp_LocalI <- output_Hosp$LocalI

HospCheck8nb$Hosp_pval <- output_Hosp$pval

HospCheck8nb$Hosp_quad <- output_Hosp$quad

write.csv(HospCheck8nb, file="~/Documents/Data/Checking8nb_Hosp.csv")

- **Run spatial models, while accounting for spatial random effect**

NOTE: Leroux models are used in this study, however, other random effect models can be tested (Reference: Lee 2013, Journal of Statistical Software)

### Run spatial models

Prev.SP <- S.CARleroux(formula= formula.P, data= prev.data, family="poisson", W=W.mat, burnin=100000, n.sample=500000)

Phys.SP <- S.CARleroux(formula= formula.P, data= Phys.data, family="poisson", W=W.mat, burnin=100000, n.sample=500000)

ED.SP <- S.CARleroux(formula= formula.P, data= ed.data, family="poisson", W=W.mat, burnin=100000, n.sample=500000)

Hosp.SP <- S.CARleroux(formula= formula.P, data= hosp.data, family="poisson", W=W.mat, burnin=100000, n.sample=500000)

## NOTE: start with shorter iterations first and check convergence, then increase as needed

## Visually check convergence

plot(Prev.SP$samples$rho)

plot(Phys.SP$samples$rho)

plot(ED.SP$samples$rho)

plot(Hosp.SP$samples$rho)

## Print results

print(Prev.SP)

print(Phys.SP)

print(ED.SP)

print(Hosp.SP)

## Export outputs

write.csv(Prev.SP$summary.results, file="SPresults_Prev.csv")

write.csv(Phys.SP$summary.results, file="SPresults_Phys.csv")

write.csv(ED.SP$summary.results, file="SPresults_ED.csv")

write.csv(Hosp.SP$summary.results, file="SPresults_Hosp.csv")

- **Run models, without presence of spatial random effect (rho=0)**

We run models without presence of spatial random effect to check whether random effect has a counfounding effect on the results.

### Run models, without random effects

Prev.noRE <- S.CARleroux(formula= formula.P, data= prev.data, rho=0, family="poisson", W=W.mat, burnin=100000, n.sample=500000)

Phys.noRE <- S.CARleroux(formula= formula.P, data= Phys.data, rho=0, family="poisson", W=W.mat, burnin=100000, n.sample=500000)

ED.noRE <- S.CARleroux(formula= formula.P, data= ed.data, rho=0, family="poisson", W=W.mat, burnin=100000, n.sample=500000)

Hosp.noRE <- S.CARleroux(formula= formula.P, data= hosp.data, rho=0, family="poisson", W=W.mat, burnin=100000, n.sample=500000)

## Print results

print(Prev.noRE)

print(Phys.noRE)

print(ED.noRE)

print(Hosp.noRE)
